# Navigating a Misty Road: Novel Ways to Study the Impact of Cognition on Driving Performance in Multiple Sclerosis

**DOI:** 10.3390/brainsci15091017

**Published:** 2025-09-20

**Authors:** Ioannis Nikolakakis, Panagiotis Grigoriadis, Nefeli Dimitriou, Dimitrios Parisis, Grigorios Nasios, Lambros Messinis, Christos Bakirtzis

**Affiliations:** 1 Second Department of Neurology, School of Medicine, Aristotle University of Thessaloniki, 54621 Thessaloniki, Greece; yiannisnikolakakis@gmail.com (I.N.); pgrigorn@auth.gr (P.G.); dparisis@auth.gr (D.P.); 2 Department of Speech and Language Therapy, University of Ioannina, 45332 Ioannina, Greece; nefelikdimitriou@gmail.com (N.D.); nasios@uoi.gr (G.N.); 3 Laboratory of Neuropsychology and Behavioral Neuroscience, School of Psychology, Aristotle University of Thessaloniki, 54124 Thessaloniki, Greece; lmessinis@psy.auth.gr

**Keywords:** cognitive impairment, driving performance, multiple sclerosis, fMRI, p300, driving simulator

## Abstract

**Background/Objectives**: The ability to drive is closely linked to participation in daily activities and quality of life in people living with neurological disorders. Cognitive deficits in people with multiple sclerosis (pwMS) are known to hinder this ability, yet concrete fitness-to-drive criteria remain elusive and assessment guidelines lack uniformity. A plethora of cognitive tests have provided associations with various aspects of driving performance and on-road behavior; however, several studies reveal limitations and inconsistencies in most tests’ sensitivity and predictive effect. Novel and resurfaced modalities for cognitive assessment, in the form of advanced imaging techniques and electrophysiological studies, may offer improved sensitivity in driving-related abilities in earlier and milder stages. Their application in addition to evaluations in driving simulators may aid future research and enhance the quality of evidence to inform decision-making. **Methods**: We searched for the relevant literature in the PubMed database and synthesized the available findings for the applications of currently clinically used cognitive tests, markers derived from functional magnetic resonance imaging (fMRI) and diffuse tensor imaging (DTI), as well as event-related potentials (ERP). **Results**: Advanced imaging modalities and ERP studies may better capture neurobiological changes that lead to driving impairment in pwMS, and they may also be applied to detect cognitive alterations earlier and with greater precision, helping to predict driving difficulties in this population. **Conclusions**: Novel tools and driving simulator settings could improve our understanding of the relation between cognition and driving in pwMS, enhance protocol homogeneity in driving studies, and aid in the formation of guidelines. The evidence in this review supports an increase in their application in future studies.

## 1. Introduction

Multiple sclerosis (MS) is a chronic, multi-focal, immune mediated, inflammatory demyelinating, and neurodegenerative disorder affecting the central nervous system (CNS) that can potentially interfere with any function [1]. Impairments in mobility, vision, and cognition are expected to hinder an individual’s ability to navigate on the road safely. Multiple sclerosis preferentially affects young individuals [2], increasing the overall burden caused by disease-related factors that interfere with the ability to drive among socially and professionally active individuals. Concomitantly, in children and young adults (ages 5–29 years old), road traffic injuries are ranked as the primary cause of mortality as per the 2018 WHO report [3]. Furthermore, the disease course is unpredictable and highly variable between individuals. This leads to differences in type of symptoms and dysfunction, frequency of relapses and rates of progression, and in psychosocial impact which hampers efforts for prognostication and creation of sensitive predictive models, including when it comes to aspects of daily living that are linked to quality of life such as the capacity to drive. PwMS experience difficulties with daily activities, particularly when there is a high level of cognitive effort required by the relevant tasks, as is the case with driving. Approximately half of the affected individuals have been reported to develop cognitive impairment, which can be expressed as becoming easily distracted or becoming slower in processing information, and it appears to aggravate difficulties.

PwMS are reported to be at least 3 times more likely to be involved in a traffic accident leading to an emergency department evaluation than healthy individuals of the same age [4]. They may also react slower, commit an increased number of errors, and have more accidents compared to controls in a driving simulator environment [5]. There has been a surge in the research literature pertaining to the driving ability of MS patients in the last decade and there have been consistent findings supporting impairments in driving performance. However, despite substantial evidence indicating impaired driving performance, clear guidelines on driving fitness in individuals with MS remain lacking [5,6].

### 1.1. Cognitive Measurements of Driving Ability and On-Road Behavior

The increased frequency of traffic accidents in pwMS is expected to be related to overall disability. However, the finding of an elevated occurrence of accidents was not replicated in the subpopulation of pwMS without cognitive impairment [7]. A positive association was found only in the pwMS with verified deficits in cognitive measures. Early studies identified the possibility of cognitive deficits having a greater impact on driving capacity than physical limitations in pwMS [8,9,10]. Furthermore, the Expanded Disability Status Scale (EDSS) score has been shown to be insufficiently predictive of error frequency and accident rate [11,12,13]. Rather, the multidimensional Multiple Sclerosis Functional Composite (MSFC) and, notably, its component focusing on cognitive status, the Paced Auditory Serial Addition Test (PASAT) were better correlated with driving performance [8]. Later studies implicated numerous markers of cognitive function as predictors of difficulties in driving.

Executive dysfunction and impairments in divided attention, learning, and memory were related to increased crash risk in a simulated driving environment. In a study by Marcotte et al., errors in the Trial Making Test-B (TMT-B) and the Hopkins Verbal Learning Test-Revised (HTLV-R) were linked to poor lane position maintenance and inadequate response time to lead car speed changes [14]. Schultheis et al. demonstrated that reduced information processing speed and deficits in visuospatial learning and recall, as measured by the Symbol Digit Modality Test (SDMT) and 7/24 Spatial Recall Test (SPART 7/24), respectively, correlated to increased traffic violations [15]. Negative results in the Useful Field Of View (UFOV), Trial Making Test-A (TMT-A), Stroop word and color tests, the compass, direction, and Road Sign Recognition (RSR) components of the Stroke Drivers Screening Assessment (SDSA), PASAT, and an array of visual tests were moderately associated with poor performance by pwMS on a subsequent road trial [10]. These measures relate to the executive function, information processing speed, attention, working memory, visual processing, and visuospatial skills of individuals and led the authors of the study to propose a model based on the most sensitive components of those tests that included the SDSA’s direction, compass, and RSR (3 out of the original 4 parts of the test), the Stroop color test, and the Speed of Processing (SoP) test of the UFOV to predict pwMS that require further examination of their fitness-to-drive [10]. Predictive value was also assigned to Rey–Osterrieth complex figure test (ROCF) as a measure of visuospatial processing ability, Stroop color–word test as indicator of response inhibition, and visual indices of binocular acuity, vertical visual field, and stereopsis, corresponding to difficulties in different aspects of a driving test [16]. Works from Morrow et al. [17] and Akinwuntan et al. [18] added the Immediate Recall component of the Brief Visuospatial Memory Test-Revised (BVMTR-IR) relating to visuospatial memory and the SoP part of the UFOV test relating to visual information processing, respectively, as predictors of on-road performance. Krasniuk et al. identified the delayed recall part of the BVMTR (BVMTR-DR) for its correlation with speed regulation and lane maintenance errors in an on-road specialized maneuver test, designed to evaluate the impact of cognitive deficits [19]. Harand et al. showed that individuals with MS struggle to maintain speed and trajectory, a result that they attributed to possible attentional deficits. The neuropsychological tests used in their study (alertness and attention tests of the TAP Battery) are commonly employed in clinical practice and failed to identify significant reduction in corresponding performance, leading to the conclusion that traditional cognitive measures may be inadequate to reveal dysfunction in alertness and divided attention [20]. Increased reaction time and mistakes in the subparts of the Test battery for attentiveness testing for Mobility (TAP-M) and impairment in visuospatial short-term and working memory, evidenced by the Wechsler Block-Tapping test forward and backward, correlated to a higher number of driving errors and accidents in a driving simulation scenario [5]. The discrepancies in the TAP-M are considered measures of diminished alertness, attention, and vigilance. The people participating in the investigation exhibited only mild impairment and the authors hypothesized that the applied neuropsychological tools might not be sufficiently sensitive to identify changes at this stage of the disease. In spite of the good functional status, all pwMS performed poorer on cognitive tests evaluating executive function, information processing, visuospatial skills, alertness, attention (visual, selective, and divided) memory, and fatigue than the control group [5].

A systematic review containing the aforementioned studies found significant methodological heterogeneity and contradictory results for many of the cognitive measures that had been exhibited previously as capable of informing predictions on aspects of driving performance and behavior. Only two tests, the SDSA and the UFOV, were consistently able to assess the effect of alterations in specific cognitive domains on the ability to drive [12]. Performance in the BVMT-R test frequently presented correlation as well, albeit less consistently among studies, while visual acuity was the non-cognitive marker with the most important impact. A similar earlier review also identified the SDSA and the UFOV as having enough supporting evidence to exhibit a likely predictive value of fitness-to-drive status. Other tests such as the TMT, BVMT, and SDMT were deemed only likely able to inform predictions of driving performance, as did visual acuity. The measures of physical disability were shown to likely not predict on-road difficulties and the authors proposed that, in pwMS without severe disability, a prioritization of cognitive and visual assessment should be considered [21]. Contradicting findings have additionally been noted with regard to early stages of the disease and in pwMS with no or mild disability [15,22].

Depression, anxiety, chronic pain, and fatigue are common in pwMS and are expected to affect driving performance. Most studies include measurements of depression, anxiety, and fatigue as well as statistical enquiries on their potential impact [10,13,16,22]. However, the synthesis of the results of relevant studies did not demonstrate a significant association with driving errors and unsafe driving behavior [12]. Amongst them, fatigue, ranging from mild to severe enough to restrict one’s participation in daily activities, would be the likeliest culprit of worse driving ability by affecting both motor and cognitive performance. However, pwMS with fatigue did not perform worse than controls and fatigue did not affect driving metrics in a simulator study by Seddiq Zai et al. [5]. On-road testing for longer duration did not reveal a significant impact either [10].

The aforementioned studies date from 2002 up to 2024, some of which date before the turn of the century. Expectedly, significant methodological and data heterogeneity originate from this lengthy time span, in addition to advances in the field and changes in nomenclature. Early studies did not report on participants’ MS phenotypes [4,8] and phenotype reporting became progressively more thorough; yet, studies either did not look for possible correlation or were not designed to investigate for statistically significant association [16,18,22]. Remitting-relapsing MS (RRMS) represents the major group of participants, with RRMS individuals frequently constituting 80% or more of the pwMS study population [13,18]. This reflects the overall phenotype distribution of the disease [1], but a lack of representation and inadequate focus on the effect of MS subtype on cognitive measurements and driving performance might lead to important omissions and restrict generalizability of the findings. Most studies investigating the relation between alterations in cognition and fitness-to-drive also elect to exclude pwMS with high disability scores on the EDSS [17], commonly citing physical restrictions as a potential unwanted confounder [8]. However, the few studies that included pwMS with higher EDSS scores found similar results concerning cognitive test results and difficulties in driving [14,18], hinting towards the complexity of each factor’s impact on overall performance and the need for enrolling participants of all levels of disability, as well as applying other modes of evaluating disease burden such as the MSFC in future studies [13]. Reviews and meta-analyses on the subject suffer from significant data heterogeneity in addition to the limitations of the cognitive tests that are traditionally used in clinical practice and research for the evaluation of pwMS. Basic demographic characteristics and key findings of the studies discussed in this section can be found in Supplementary Table S1.

### 1.2. Limitations of Traditional Tests from the Neuropsychological Evaluation

The studied population of pwMS performs consistently worse in approximation tests, simulations, or direct on-road assessments compared to healthy controls [5,13,14,15,20,23,24]. Official records and registries support that people with neurodegenerative and/or neuroinflammatory disorders drive worse [7,25]. Contrary to these statements, the majority of the neuropsychological metrics that have been identified as able to predict impairments in driving performance are not consistent across studies [12], exhibit conflicting results [21], or are not sensitive at different disease stages or disability levels [13,20,22]. It is likely that they are limited in assessing driving-altering cognitive deficits due to overlaps [26], blind spots, and due to an inability to detect early neuronal changes. The role of brain reserve and compensatory behaviors should also be taken into account for the dissociation between evident driving strains and normal cognitive metrics on traditional evaluation. Cognitive reserve has been positively correlated to memory functions, related to different modalities and postulated to account for radiological-clinical dissociation in assessment of cognitive status [27]. Thus, it might impose another limitation on those metrics. Finally, social cognition is expected to affect driving behavior since on-road transport involves constant interaction with multiple other vehicle users. However, social cognitive factors that are expected to participate in crash-risk estimation, such as Theory of Mind (ToM) and empathy, have been sparsely considered in the literature [28]. Empathy, overall ToM, and cognitive ToM have been found to be impaired in pwMS with remitting-relapsing and progressive phenotypes in relation to healthy controls [29]. Healthy individuals that performed worse in a task evaluating ToM also exhibited a higher reaction time to hazardous situations, highlighting ToM capacity as a potential predictive aspect for an individual’s risk of driving accident [29]. Routine neuropsychological evaluation of pwMS frequently does not employ dedicated tests for social-cognitive aspects, as evidenced by their absence in the studies of the previous section, thus limiting the potential of forming relevant observation. Currently, facial emotional recognition tasks and measurements are being applied to fill these gaps.

Adding to their pitfalls, studies applying cognitive tests to evaluate the impact of frequent MS comorbidities on driving performance failed to identify significant associations. However, studies examining the effect of these diseases directly have found a correlation to unsafe driving behavior [30,31]. This corroborates a lack of sensitivity rather than a lack of relation. A possible explanation may be the inadequate ability to uncover underlying specific network dysfunction. For instance, it has been hypothesized that pwMS drive safer and perform better in environments that offer higher levels of stimulation [22]. Likewise, pwMS committed more driving errors in a monotonous setting under good weather conditions in a simulator test, indicating a possible deficit in arousal [11]. These findings implicate dysfunction in the attention networks and arousal network as a probable neurobiological basis of the link between cognitive impairment and poor driving performance. Indeed, reduced vigilance caused by prolonged monotonous driving led to reductions in reaction time in controls, especially older ones [32]. PwMS had more symptoms of daytime sleepiness towards the end of a monotonous drive, hinting to a resulting state of hypovigilance by such conditions [33].

Imaging modalities and electrophysiological measurements may be more sensitive to the structural and functional substrate of changes in relevant cognitive domain. Advanced magnetic resonance techniques, namely DTI and fMRI and ERPs stand out as refined tools for the evaluation of executive function, alertness, attention, processing speed, and mental flexibility [34,35]. A synthesis of the literature to substantiate their application for this purpose is presented below.

## 2. Functional MRI and Diffusor Tensor Imaging

In a complex and dynamic task such as driving, the requirement of high cognitive load and continuous interaction of networks that exert top-down control and bottom-up feedback is expected in order to meet demands. Functional MRI has been a revolutionary tool in the study of functional dynamic processes in the brain, including network connectivity and activation. Resting state fMRI (RS-fMRI), visualizing spontaneous activity, and task-based fMRI have shed light on the activity underlying cognitive functions in healthy and pathological conditions [36]. DTI is another advanced MRI technique that utilizes diffusion measures to visualize the microstructure and topography of white matter. It has offered the ability to evaluate white matter pathways in vivo and the information about network connectivity acquired by RS-fMRI has been evidenced to largely relate to DTI structural connectivity [37]. The combination of these methods has been used effectively to enhance our understanding of normal processes like aging and neurological disorders such as amyotrophic lateral sclerosis and progressive supranuclear palsy [38]. Moreover, findings support the capacity of RS-fMRI to predict task-dependent activation of intrinsic networks by estimating functional connectivity of those networks during rest. A large-scale mechanism of activity flow was postulated as a link between similarities in patterns of RS and cognitive task-based functional connectivity [39]. This is highly pertinent to informing predictions about complex skills such as driving, which are labor- and resource-intensive, to reproduce in a magnetic scanner setting. It also eliminates the need to perform a complex task that would otherwise impede compliance of pwMS with significant disability, which is a population of interest for such evaluations [40].

On a theoretical level, demyelination and white matter damage are expected to decrease efficiency of signal transmission and slow down information shifting and processing. Large scale communication, as well as on a subnetwork level, is thought to depend on integrity and navigational efficiency of white matter topology, a more preserved one allowing for an efficient execution of high-order cognitive tasks [41]. Additionally, an overall whole-network efficiency reduction appears to consistently signify cognitive impairment in pwMS [42]. Hence, the functional collapse of networks and global topological disruption, as demonstrated by structural imaging (e.g., tractography by diffusor tensor imaging) and fMRI, may identify at a high-rate cognitively impaired individuals [41,42]. Their utility includes informing about disease and disability stage, as the earlier findings consist of diminished long-range connections and a compensatory increase in local efficiency and activation which progresses with increasing structural damage to hypoactivation and loss of network efficiency [40].

DTI and fMRI are capable of revealing functional and structural connectivity disorders of specific networks in MS. Based on steady state along with connection and activation patterns, networks involved in cognitive functions that have been already mentioned as crucial for driving performance can be analyzed. The main networks are the default mode network (DMN), the fronto-parietal attention network (FPAN), the ventral attention or salience network, the dorsal attention network (DAN), the sensorimotor (SMN), and the alertness network. A large fMRI study of pwMS indicated that cognitive impairment was primarily correlated to a decreased degree centrality of the salience network and increased connectivity of the DMN. It also demonstrated that sensorimotor hub weakening takes place compared to healthy controls. Both DMN and sensorimotor networks are interconnected to the salience network, which led Carotenuto et al. to postulate that salience dysfunction in MS compromises shifting and integration between internal and external stimuli and hinders pwMS’ performance in cognitive and complex tasks [43]. Consequently, the association of degree centrality measurements with impairments in cognition and functional status highlights their potential use as quantitative markers in this population.

Furthermore, decreased structural integrity and altered network dynamics have been demonstrated in pwMS and associated with dysexecutive function, attentional deficits, and decreases in information processing speed. Eijlers et al. employed fMRI measures and showed reduced functional connectivity of DMN and front-parietal attention network in addition to the loss in dynamic interplay of DMN and visual networks. This contrasts to their expected opposed activation in cognitively impaired participants with MS. Healthy controls and pwMS without cognitive impairment did not demonstrate similar findings [44]. Moreover, reductions in fractal anisotropy alluding to weakened structural connectivity of the fronto-parietal network and the insula in pwMS correlated to worse performance in the PASAT and SDMT. An increase in fractal anisotropy of connections from frontal, cingulate, and occipital cortices was also noted, suggesting an early compensatory structural reorganization in response to cerebral damage [45].

The FPAN has been implicated in defining processing speed in both cognitively demanding assignments and automatic processes in healthy controls studied by tractography [41]. The ability of the FPAN to shift states between rest and performing an assignment, measured in changes in static and dynamic functional connectivity, has also been linked to performance of pwMS in attentional tests. An elevated FPAN dynamic connectivity at rest may predict difficulties in tasks requiring sustained attention [46]. DMN has been implicated in the processing of information relating to internal state and is activated during internal mentation and mind-wandering, thus a reduction in its activation is thought to be taking place in activities requiring focus on external stimuli. Contrary to this, recent findings suggest a reconfiguration instead of a downregulation of its connectivity during tasks and showed a positive relation between normal function of DMN and processing speed, measured by reaction times in a finger opposition assignment [47]. The relation between the connectivity of these two networks has been noted as highly pertinent to cognition in pwMS and is thought to be mediated by the salience network. Longitudinal fMRI studies hint towards an initial overload of the salience system and a progression to dysregulation of the DMN and FPAN in cognitive impaired individuals [40].

Finally, studies of neural circuits during actual driving established a relationship between network connectivity patterns and driving behavior. Concerning the driver’s attentional state, interconnectivity of the DAN and VAN, as well as functional connectivity between DAN and SMN, were demonstrated to reflect the level of distraction [48]. In addition, altered global connectivity of the lateral prefrontal cortex and a reduced connectivity within the SMN and between DAN and FPAN during a virtual real-world task has been shown to predict worse multitasking performance that may affect driving ability [49].

### 2.1. Event Related Potentials

Event-related potentials (ERP) are an electrophysiological modality that has re-emerged thanks to technique refinement as a valuable tool for the evaluation of cognition. They are produced in a similar fashion as other evoked potentials by using scalp electrodes and calculating through time the response to stimuli of various nature (cognitive, tactile, visual, auditory; the auditory and visual oddball paradigms being the most frequently used in the literature) [50]. Several components of the produced ERPs have been used in clinical practice and the P300 has been the most thoroughly studied. The DMN is an important generator of the wave and alterations in P300 amplitude and latency have been shown to correlate to poorer cognitive performance in neuropsychological tests [51]. Notably, prolonged P300 is associated with slower information processing speed, while lower P300 amplitude is thought to correspond to disruption in frontal, parietal, and subcortical (notably, the thalamus) networks and reduced attentional resource allocation. Both have been demonstrated to be a frequent finding in pwMS, even in the setting of normal performance in established cognitive tests such as SDMT and PASAT [51,52]. Evidence provided by studies in pwMS also linked P300 abnormalities to lower levels of educational attainment, implicating the role of cognitive reserve, and ERPs are considered a valuable tool for detecting alterations in compensatory mechanisms prior to the emergence of apparent cognitive impairment [50,52]. The N100 is a less well-studied wave that informs on perceptual and attentional processes and about perceptual resource allocation [53]. These two EPR indices, similarly to fMRI and DTI measurements, offer the advantage of a more direct association to underlying physiological and abnormal processes in comparison to the neuropsychological tests (Table 1).

ERPs allow for monitoring cognitive loads amidst driving and offer great temporal precision for the evaluation of the way a driver processes information, interacts with traffic and reacts to a stimulus. P300 reduction in amplitude and latency prolongation are associated with mind wandering and distraction and can reflect the level of cognitive fatigue in drivers. In healthy individuals, these alterations in P300 amplitude and latency were reported in the presence of increased driver-related risk, were suggested to reveal suboptimal information processing and cognitive function, but did not lead to significant decrements in driving performance, possibly due to compensation and additional neural network recruitment [35]. P300 and N1 prolonged latency and lower amplitude specify increased mental workload demands in complex tasks. Additionally, prolonged simulated driving in monotonous conditions led to slower reaction times and reduced P300 amplitude towards the end of the 4 h task, underlining the relation of the amplitude to vigilance and alertness and a possible connection to unsafe driving [32]. N100 and P300 in the Solis et al. 2018 study were the sole indices capturing the elevation in cognitive load that was associated with increased demand on processing speed in dual and triple tasks that were used to represent real-life complex-task behavior [53].

Measurements based on evoked potentials have also proven their ability to detect subclinical threshold changes in other functional systems. PwMS frequently present involvement of the visual system as part of acute relapses or silent disease progression. Results from the application of visual evoked potentials, optical coherence tomography of the retinal nerve fiber layer and the macula, as well as other visual tests (i.e., visual field test and standard achromatic perimetry and contrast sensitivity test), suggest that a significant portion of pwMS reporting no visual symptoms harbor subclinical visual impairment [54,55]. Such impairment may alter visual information processing speed, visual attention, and spatial awareness, leading to unsafe driving behavior. The aspect of visuospatial learning and recall has also been substantiated in predicting driving capacity [15].

### 2.2. Virtual Reality Environments and Driving Simulator Testing

Another caveat concerning the studies focusing on the driving ability of pwMS and the inconsistencies in their findings pertains to the preferred method used for driving assessment. Broadly, four types have been used in the literature: (a) traffic violations and accidents reported in official driving records and agencies archives; (b) computerized measures of skills pertaining to driving; (c) driving simulation tests; and (d) on-road tasks and assessment (Table 2). Self-reporting questionnaires were also used sparsely in the literature in combination with other modes. Even though on-site road assessment is perceived as the standard by which all other measures are compared against, there are controversies regarding its ability to definitively resolve the question of one’s fitness-to-drive. It introduces biases, namely due to the driving instructor, time of day, weather, and road conditions, and is limited by safety restrictions and physical constraints.

A relative shift in the applied method has taken place over time. Initial studies relied on driving reports and data from official agencies as an extrapolation of a driver’s ability [4,7]. Later on, metrics from adapted computerized tests, such as Useful Field of View and the Neurocognitive Driving Test, were more prevalently applied [8,10]. As the literature on the subject advanced and the hypothesis formulation became more sophisticated, advances in technological instruments were utilized to perform simulator-based studies to mimic the dynamic and highly variable nature of a task such as driving. Their use for assessment in pwMS has been demonstrated to be feasible and efficient in specific subgroups [56]. Simulated scenarios have been successfully applied to demonstrate the impact of spasticity, deficits in attention and vigilance, and overall cognitive impairment in pwMS [11,14,20]. Driving simulators offer a greater opportunity for standardized replicable protocols for comparable outcomes. Yet, machinery and protocols varied in earlier studies and favored only static simulators [56]. They also stand to be further enhanced by virtual reality applications and construct more demanding or precise challenges [20,22]. In a recent study by Pierella et al., the virtual reality driving environment was well received by participants and could elicit realistic behaviors and differences between pwMS and controls. The simulator outcomes in the MS group indicated deficits in lane maintenance and adjustment to stimuli [57]. Devos et al. supported the evaluation of risk-related behaviors such as real-time daytime sleepiness as possibly being more ecologically sound in a simulator environment. In their study, pwMS exhibited aggravated symptoms of sleepiness towards the end of a monotonous drive by their objective metric of choice (percentage of eyelid closure), while reported sleepiness failed to differentiate them from controls [33]. This is among the first evidence of adapted measures where driving simulators perform better than traditional tests. Automated tools have been developed to grade driving performance in a simulator and have been applied in judging ability to resume driving post-brain injury [58]. In this study, worse grades of D and E produced by the automated tool were significantly higher in the MS group and a D or E grade was associated with worse performance in the simulation, as did a lower SDMT score [58]. This evaluation protocol, combining neuropsychological tests and driving simulator measurements that can be automated and quantitated, may offer a possible basis for future studies aiming to develop decision-making tools.

There is still no consensus on the best method to capture the multifaceted and dynamic aspects of driving, and a driving simulator environment seems the most able to adopt both novel techniques of evaluation as well as traditional cognitive tests. Adjustments may be made to neuropsychological measures for them to better fit a driving simulator testing setting and potentially increase their sensitivity. Alongside such developments, the prospect of portable driving simulators has been tested and was preliminary found to be user friendly and efficient in a driving rehabilitation study [59], standing to increase their accessibility.

Driving performance and behavior measured by traditional or novel tests in simulated complex and demanding tasks in changing conditions, that in a physical setting could pose significant safety dangers, could increase the precision of the evaluation. Likewise, in a driving simulator, a more personalized environment can be attained for driving rehabilitation, with different levels of difficulty and scenarios fitted to the specific deficits noted in each individual. Even though there is a paucity of evidence regarding their application in rehabilitation, a 2014 pilot study demonstrated a trend toward greater improvement in visual, motor, and cognitive measures as well as in fatigue after training in a simulator for 5 h over 5 weeks in comparison to controls that received no training [60]. Extrapolating from data of stroke patients, such a setting offers the opportunity to practice driving skills and develop compensatory strategies in more accessible and safe environments, which is especially promising for individuals that are anxious about using a vehicle or have developed negative beliefs after new or progressive neurological deficits. Furthermore, an important advantage stands to be the prospect of informing patient perception, helping individuals that are repeatedly experiencing significant difficulties in the simulator understand their limitations and accept their inability to resume driving [61]. This can enhance participation and compliance with a rehabilitation program. However, there is still inadequate evidence to suggest long term positive effects and data on pwMS is lacking [56,62]; thus, the role of driving simulators should remain as an adjunct and a supplement of educational programs and on-road training until further research on the subject is concluded. Altogether, the application of driving simulators offers promising advantages for both research and decision-making purposes and represents a novel field for driving rehabilitation.

## 3. Discussion

Cognitive impairment in MS affects individual driving capacity, starting from the very early stages of the disease. It appears to be as important a factor as mobility and vision and possibly even more important in the group of pwMS with mild or moderate disability in informing predictions about driving [7,12,14].

This review reiterates that pwMS experience greater difficulties with driving compared to healthy controls, yet it remains uncertain to what degree MS-related cognitive dysfunction alters driving performance and contributes to these challenges. Many of the regularly used neuropsychological tests appear not to be sensitive enough in early disease stages [22], for individuals with mild impairments [5], perhaps due to compensatory brain and cognitive reserve mechanisms. Thus, employing novel measures to identify different factors that affect driving ability may increase sensitivity and elucidate new aspects of this issue. Application of such tools is crucial to respond to the lack of established criteria for fitness-to-drive in pwMS. Future studies may additionally benefit by using them as adjuncts for evaluating the effect of these understudied contributing factors, such as cognitive reserve and impairment of social cognition.

**Figure 1:**
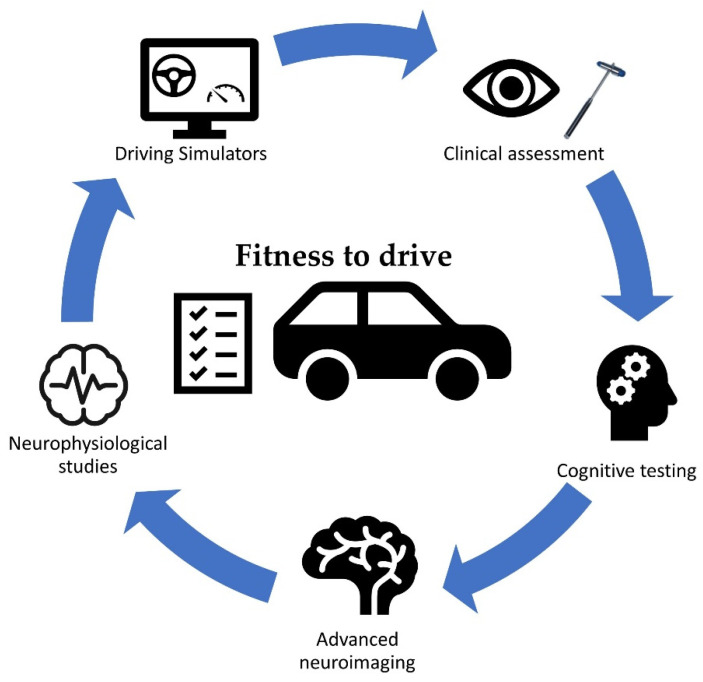
Possible application of novel ways for evaluating cognitive impairment and testing driving ability from research to practice. Besides routine clinical and cognitive examination, neuroimaging and neurophysiological studies may further enhance our understanding of dysregulation of networks and brain connectivity and the way in which they relate to cognitive impairment and poor driving performance in pwMS. An increased utilization of driving simulator environments in studies directly evaluating driving behavior in pwMS stands to homogenize protocols and increase task complexity and difficulty. All together may enable an enhanced prediction of fitness-to-drive.

Advanced imaging techniques stand to help on this front by directly assessing the underlying networks that form the neurobiological basis of cognitive functions. DTI and fMRI evaluate different aspects of brain connectivity and, in combination, have been already applied in the study of the pathophysiology of neurological disorders. Dysregulation of large-scale integration and local segregation harboring diminished network efficiency is associated with cognitive impairment in pwMS [63]. FMRI and DTI are able to visualize damage in specific brain regions and strategic white matter tracts leading to widespread underlying disconnection that may better account for cognitive dysfunction than lesion load and diffuse abnormalities [64]. The high cost and need for specialized equipment remains however a significant limitation for clinical application. Cost-effective and objective evaluation of cognitive status and task-related cognitive responses is feasible by the utilization of ERP measures. Indices derived by their application have been shown to be able to predict increased driving risk, the latency and amplitude of the well-studied P300 wave being the most notable among them [35]. Excellent correlation to information processing speed and aspects of attention has been demonstrated and an earlier identification of deficits by their application seems possible [50]. Both tools exhibit the significant benefit of directly correlating to neurobiological substrates that offer them the ability to add to clinical and neuropsychological measures. Their application can aid the physician to identify early and accurately individuals that are at risk of declining driving ability and require institutional rehabilitation training or further assessment (Figure 1).

A caveat of driving studies is methodological heterogeneity. Various protocols and different measures of outcome assessment have been applied [12]. The exclusion of pwMS with higher EDSS scores and modest investigation into how disease phenotypes impact on driving ability are a limitation that should also be addressed in future studies. The use of a driving simulator environment has shown promise as a reliable alternative to on-road assessment [56]. Standardized driving simulator protocols offer a great opportunity for replicable and complete testing of driving scenarios with comparable measurements. Driving simulation and virtual reality devices have been proposed as capable of creating more engaging and demanding conditions in an effort to uncover subtle changes in cognitive function, notably in the domains of alertness, attention, and information processing, that real-life road scenarios may be unable to mimic due to safety concerns [20,21]. Furthermore, commonly utilized tests, such as the PASAT and SDMT, showed early promise but reviews on the subject revealed limitations in their performance as predictors of on-road behavior and fitness-to-drive. A reason for their limited predictive value may relate to testing in physical conditions. The application of simulated scenarios may present a second wind for the traditional cognitive tests. Their combination and adaptation to such settings may help increase sensitivity [58]. Simulated driving is also feasible inside a magnetic scanner and during electrophysiological studies. Moreover, automated grading tools and portable simulators can augment their applicability and accessibility [59]. Thus, driving simulators offer methodological advantages for future studies and are a promising alternative with unique qualities for decision-making guidelines and assessments. Finally, more studies should focus on their role and efficacy in driving rehabilitation. Driving simulators present significant benefits for such purposes, including increased safety, accessibility, and a greater capacity for personalization, but there is currently very limited data in the population of pwMS.

The privilege of driving is vital for the majority of pwMS to aid them in safeguarding their social, professional, and daily life. Revocation of driving privilege could lead to functional decline by direct and indirect effects, such as the inability to participate in rehabilitation programs or assess health services. It is also expected to aggravate mood disorders and sense of isolation. There is a delicate balance between the quality of life and needs of pwMS and others’ safety during transport and forming criteria and guidelines is challenging, as the margin for error is very narrow. For this purpose, longitudinal studies are needed that will incorporate novel findings and assessment methods in the evaluation of MS impact on driving performance. It might also be beneficial to investigate how imaging markers and ERP indices track disease and cognitive decline in relation to driving ability.

While waiting for a firm establishment of fitness-to-drive criteria for pwMS and assessment guidelines harmonization, it appears prudent to follow a similar pattern of frequent evaluation, as is recommended in cognitively compromised individuals. Recent recommendation on cognitive evaluation endorses annual testing with validated instruments and recommends proceeding to more comprehensive assessment in cases of impaired performance [65]. Likewise, pwMS who are at risk (i.e., exhibiting motor or cognitive impairment or reporting alterations in driving) may benefit from annual assessment with traditional and, potentially in conjunction with, novel tools and poor performers should be referred for formal on-road evaluation of fitness-to-drive. Adaptive neuronal plasticity is more prominent in the initial course of the disease [66]; thus, early recognition offers the chance of timely rehabilitation and implementation of preventive measures, such as vehicle modification.

Finally, the increasing need for frequent evaluation and the vital role of early cognitive rehabilitation require pwMS to travel to specialized facilities despite their driving difficulties—a problem that is aggravated in the case of residents of rural areas and individuals with limited support systems. Virtual reality tools and simulator environments may help once more by increasing accessibility and transportability at remote sites, ensuring adherence to protocol standards and specialized supervision.

## 4. Conclusions

Cognitive impairment in pwMS is a significant factor in declining driving ability, together with deficits in motion and vision. Neuropsychological tests have been the basis of assessing the relationship between cognitive status and driving performance, but they exhibit significant limitations that restrict their sensitivity. Tools such as fMRI, DTI, and ERPs could enhance our understanding of the neurobiological basis of driving impairment in pwMS. More studies applying novel methodologies of cognitive assessment in relation to driving performance are needed. A standardized protocol is vital for meaningful comparisons, and the use of driving simulators may be a better fit for such purposes. Their application can also create demanding tasks and increase cognitive engagement while maintaining safety. In light of the limitation of the currently employed neuropsychological assessments, novel measures and settings for evaluation should receive greater attention in future studies.

## Figures and Tables

**Table 1:** Neurobiological correlates cognitive assessment by the application of novel modalities.

Modality	Measures	Biological Correlates
Functional MRI [36,37,40,44]	Activity, structural—functional—dynamic connectivity, path length, clustering co-efficient, degree centrality, dynamic eigenvector centrality	Integrity and segregation, modularity and centrality of relevant networks, network efficiency, dynamic fluctuation, and reorganization
Diffusion Tensor Imaging [37,38,40]	Structural connectivity, fractional anisotropy, mean diffusivity	WM integrity, fiber paths mapping, tractography
P300-ERP wave [32,35,50,51,52]	Latency and amplitude, morphology	IPS, attention, salience, arousal, vigilance, short term and working memory, possible marker of navigation efficacy of specific networks and cognitive reserve
N1-ERP wave [53]	Latency and amplitude, morphology	Perceptual IPS and stimuli analysis, perceptual resource allocation, early selective attention, cognitive load

WM: White matter, IPS: information processing speed.

**Table 2:** Assessment method preference in studies on driving in pwMS.

Utilized Method of Assessment	Study
Reported violations and traffic accidents in driving records and official archives	[4,7,8,23,55]
Computerized measures of driving ability	[8,10,16,18,19,24]
Driving simulator tests	[5,11,14,20,33,56]
On-road tasks, assessment	[10,15,16,17,18,19,24]

## Data Availability

No new data were created or analyzed in this study. Data sharing is not applicable to this article.

## Supplementary Materials

The following supporting information can be downloaded at: https://www.mdpi.com/article/10.3390/brainsci15091017/s1, Table S1: Description of studies assessing cognition and fitness to drive in multiple sclerosis. References [4,5,7,8,9,10,11,12,13,14,15,16,17,18,19,20,22,23,24] are cited in the supplementary materials.

## Abbreviations

pwMS	People with multiple sclerosis
MS	Multiple Sclerosis
fMRI	Functional magnetic resonance imaging
DTI	Diffuse tensor imaging
ERP	Event-related potentials
CNS	Central nervous system
WHO	World Health Organization
EDSS	Expanded Disability Status Scale
MSFC	Multiple Sclerosis Functional Composite
PASAT	Paced Auditory Serial Addition Test
TMT-B	Trial Making Test-B
HTLV-R	Hopkins Verbal Learning Test-Revised
SDMT	Symbol Digit Modality Test
SPART 7/24	7/24 Spatial Recall Test
UFOV	Useful Field Of View
TMT-A	Trial Making Test-A
RSR	Road sign recognition
SDSA	Stroke Drivers Screening Assessment
SoP	Speed of Processing
ROCF	Rey–Osterrieth Complex Figure test
BVMTR-IR	Brief Visuospatial Memory Test-Revised—Immediate Recall
BVMTR-DR	Brief Visuospatial Memory Test-Revised—Delayed Recall
TAPM	Test battery for attentiveness testing for Mobility
RRMS	Remitting-relapsing Multiple Sclerosis
ToM	Theory of Mind
RS-fMRI	Resting state fMRI
DMN	Default mode network
FPAN	Fronto-parietal attention network
DAN	Dorsal attention network
SMN	Sensorimotor network
WM	White matter
IPS	Information processing speed
