# Supplementary material for: Navigating a Misty Road: Novel Ways to Study the Impact of Cognition on Driving Performance in Multiple Sclerosis
Source: Brain Sci. 2025 Sep 20;15(9):1017. doi: 10.3390/brainsci15091017 (PMC12469141; doi:10.3390/brainsci15091017)
Supplement: Supplementary file 1 [file brainsci-15-01017-s001.zip › brainsci-3864217-supplementary.pdf]

Supplementary table S1. Description of studies assessing cognition and fitness to drive in multiple sclerosis

| Study | Participants' characteristics | Findings: measures correlating with driving performance |
|---|---|---|
| Lings, 2002 [4] | MS phenotype not specified, Median EDSS: not available Historical cohort study based on registries and official records | No cognitive measures were reported |
| Schultheis et al, 2002 [7] | RRMS (59%) > SPMS (7%) > PPMS (4%) and 8 pwMS with no specified phenotype Median EDSS: not available Age < 55 years-old (38-48) Participants with minimal to no physical disability | PwMS with cognitive impairment may predict increased risk of motor accident (in comparison to pwMS without cognitive deficits and healthy controls). This group also performed worse on block design and symbol digit tests, Stroop Test, TMT-A and B, PASAT |
| Shawaryn et al, 2002 [8] | MS type no specified Median EDSS: not available Ambulation subscore: 0-3, PwMS with minimal to no physical involvement were included Age, mean: 58.5 | 9-HPT and 3s PASAT of the MSFC - total latency time score |
| Kotterba et al, 2003 [11] | RRMS .Mean EDSS: 2.8 Age, mean ± SD: 45.1 ± 7.8 years | PASAT of the MSFC correlated with collision rate, EDSS exhibit no significant correlation |
| Lincoln et al, 2008 [9] | MS phenotype not specified Median EDSS: not available | SDSA Dot Cancellation and RSR - Sustained attention, AMIPB Design learning, AMIPB IP (memory and information processing) |
| Marcotte et al, 2008 [14] | MS phenotype not specified Median EDSS: 6.0 Age, mean ± standard deviation: 47.7±11.7 | TMT-B, SDMT, HVLT-R (language, memory) Cognitive impairment was related with difficulties in maintaining lane position and responding to speed changes of a leading car Spasticity was associated with impaired speed maintenance and tracking of a leading car |
| Schultheis et al, 2010 [15] | RRMS (86%) > PPMS, SPMS Mean EDSS: 3.4 Age, mean ± standard deviation: 43.24 ± 8.07 | SDMT (IPS), SPART 7/24 visuospatial skills, correlating to driving ability |
| Akinwuntan et al, 2013 [10] | RRMS Median EDSS: 3.0 Age, mean: 48.05 (standard deviation of 9.13) | 9-HPT, BD, UFOV-speed of processing, Stroop word test, TMT-A, Model of SDSA's direction, compass and RSR tests (3 of 5), Stroop Color test, UFOV-speed of processing test and selective attention, PASAT Visual tests: recovery from glare, blue and violet color perception, constrast sensitivity (moderate association) |
| Dehning et al, 2014 [14] | RRMS Mean EDSS: 2.87 EDSS < 6.0 Age: 18 – 70 years-old | COWAT (verbal fluency), Block Design (visuospatial) AVTL learning (anterograde memory) and TMT-B (executive function) MRI third ventricular width (indirect for thalamic |

| | | |
|---|---|---|
| | | atrophy) and Freq of driving violations |
| Devos et al, 2017 [16] | RRMS (89%) > PPMS,SPMS<br>Median EDSS: 4.0<br>Age, mean: 47.91 (standard deviation of 8.71) | ROCF (Visuospatial ability), Stroop color-word (response inhibition), Directions test (executive function, SDSA) binocular acuity + vertical visual field + stereopsis |
| Krasniuk et al, 2017 [19] | RRMS (63%, 22 participants) or progressive MS (13 participants)<br>Median EDSS: 2.0<br>EDSS: 0 – 4.0<br>Age: 18 – 59 years-old | SDSA and UFOV test, BVMTR-IR, BVMTR-DR performance correlated to driving errors in a strategic maneuver road task |
| Morrow et al, 2018 [17] | RRMS (61.1%) > SPMS (36.1%) > PPMS (2.8%)<br>Median EDSS: 3.0<br>EDSS: 0 – 4.0<br>Age: 18-59 years-old | Poor BVMTR-IR (visual-spatial memory) performance related to driving evaluation failure |
| Akinwuntan et al, 2018 [18] | RRMS (92.3%) >SPMS,PPMS<br>Median EDSS: 5.0<br>EDSS: 3 – 7.0<br>Age: 25-65 years-old | Square Matrix Directions, Square Matrix Compass, Road Sign Recognition (RSR), Speed of Processing (SoP) were part of a battery with increased accuracy |
| Harand et al, 2018 [20] | RRMS<br>Median EDSS: 2.0<br>Age, mean ± standard deviation: 40.36 ± 7.17 years old<br>Included less cognitively impaired patients | Impaired SDMT, no differences in TAP sub-test for alertness and divided attention<br>Virtual reality testing revealed driving difficulties in participants possibly associated to attentional deficits that the traditional tests may have missed |
| Classen et al, 2018 [24] | RRMS (57%) > PPMS,SPMS<br>Median EDSS: 2.5<br>Age: 18-59 years-old | Reduction in visual acuity and worse performance in the UFOV – visual processing speed test |
| Ottersbach et al, 2023 [22] | 19 RRMS participants, 3 CIS and 3 CIS that transitioned to RRMS during the study<br><br>Mean EDSS: 0.7 ± 0.8 at baseline, 0.7 ± 0.9 at follow-up<br>Age: 22 – 44 years-old | Psychomotor vigilance task (PVT) (revealed reductions in reaction time)<br>Alertness, sustained attention and fatigue were similar to matched healthy controls<br>No specific deficits relating to fitness to drive in pwMS at one-year follow-up |
| Seddiq Zai et al, 2024 [5] | RRMS (69.1%) > PPMS (14.4%) > SPMS (14.4%) and 10 participants' type was unclear (10.3%)<br><br>Median EDSS: 2.5<br>Age: mean age 38.96 (standard deviation of 11.1) | A better WMS-R block span backwards score (working memory) was associated to more accidents<br>TAP-M visual scanning was negatively correlated to mean reaction time and number of errors, TAP-M go-no-go and WMR-S positively correlated to number of accidents<br>BVMT-R correlated to number of accidents<br>PwMS, including those at an early disease stage, had reduced reaction times and more accidents. |
| Martines-Gines et al, 2024 [13] | RRMS (27 participants, 90%)> PPMS (6.7%), SPMS (3.3%)<br>Mean EDSS: 2.4<br>Mean age ± standard variation: 35.6 years ± 8.0 | MSFC, EDSS related to 2HAND (concentration and visuomotor coordination) and speed anticipation test (SART, a computerized test used to assess sustained attention and response inhibition) performance (both driving tests).<br>PASAT and 2HAND correlation<br>MSFC may exhibit advantages to EDSS in evaluating |

| | | |
|---|---|---|
| | | driving ability |
| Krasniuk et al, 2019 [19] | Systematic review, 15 studies<br>Relapsing (76%) or progressive MS<br>EDSS: 0 – 6.5<br>Age: at least 18 years-old | Level B evidence for predicting fitness to drive: SDSA, UFOV test (may probably predict)<br>Level C evidence: BVMTR-R, SDMT, PASAT, visual evaluation of acuity |
| Seddiq Zai et al, 2022 [12] | Systematic review, 24 studies<br>RRMS (439 participants) > PPMS (29) > SPMS (25) and 13 participants unspecified<br>EDSS: range between studies was reported, from 1.95 (lowest mean) to 6.0 (highest median)<br>Age: 35.6 – 55 years-old | Visual and cognitive impairment exhibit major impact on driving performance<br>SoP of UFOV test correlated with driving ability, SDSA performance and visual acuity showed significant impact<br>Small participant samples and significant result heterogeneity |

AMIPB (Adult Memory and Information Processing Battery, MSFC: Multiple Sclerosis Functional Composite, IPS: Information processing speed, HVTL-R: Hopkins Verbal Learning Test-Revised, WMS-R: Wechsler Memory Scale-Revised) 9-HPT: 9-Hole peg test PASAT: Paced Auditory Serial Addition Test, TMT-B: Trial Making Test-B, UFOV: Useful Field Of View, TMT-A: Trial Making Test-A, RSR: Road sign recognition, SDSA: Stroke Drivers Screening Assessment, BVMT -R: Brief Visuospatial Memory Test – Revised, SDMT: Symbol Digit Modality TestSART: Sustained Attention to Response Task, WAIS-III: Wechsler Adult Intelligence Scale -Third Edition, COWAT: Controlled Oral Word Association Test, ROCF: Rey-Osterrieth Complex Figure, AVLT: Auditory Verbal Learning Test, WMR-S: Wechsler Block-Tapping test, RSR: Road sign recognition, SoP: Speed of Processing, EDSS: Expanded Disability Status Scale, RRMS: Remitting-relapsing Multiple Sclerosis, SPMS: Secondary progressive Multiple Sclerosis, PPMS: Primary Progressive Multiple Sclerosis
